# Mechanochemical synthesis and transformation of the polymorphic double carbonates fairchildite and buetschliite, (K_2_Ca(CO_3_)_2_): an *in situ* X-ray powder diffraction study†

**DOI:** 10.1039/d4mr00093e

**Published:** 2024-11-28

**Authors:** Volker Kahlenberg, Doris E. Braun, Wolfgang Schmidt, Hang Liu, Sebastian Leiting, Claudia Weidenthaler

**Affiliations:** a University of Innsbruck, Institute of Mineralogy and Petrography Innrain 52 A-6020 Innsbruck Austria; b University of Innsbruck, Institute of Pharmacy, Pharmaceutical Technology Innrain 52c A-6020 Innsbruck Austria; c Max-Planck-Institut für Kohlenforschung Kaiser-Wilhelm-Platz 1 D-45470 Mülheim an der Ruhr Germany weidenthaler@mpi-muelheim.mpg.de +49 (0)208 306 2181

## Abstract

This study presents the mechanochemical synthesis of the two K_2_Ca(CO_3_)_2_ polymorphs, fairchildite and buetschliite, from CaCO_3_ and K_2_CO_3_ using a shaker mill. Unlike previous methods requiring high temperatures and prolonged heating, fairchildite, a high-temperature polymorph, is formed initially in all experiments, adhering to Ostwald's rule of stages. Notably, the transformation to the stable buetschliite phase can be achieved by varying milling parameters, particularly frequency and moisture content. The results suggest that pressure, rather than temperature, plays a significant role in this phase transition, with moisture further accelerating the transformation. These findings offer a new, efficient route for the synthesis of these polymorphs, highlighting the critical influence of milling conditions on the reaction pathway.

## Abstract 2

The synthesis of double carbonates (K_2_Ca(CO_3_)_2_) has been studied by *in situ* and *ex situ* powder diffraction experiments. The results provide insights into the formation of these materials, which are of interest in geosciences and applied mineralogy.

## Graphical abstract

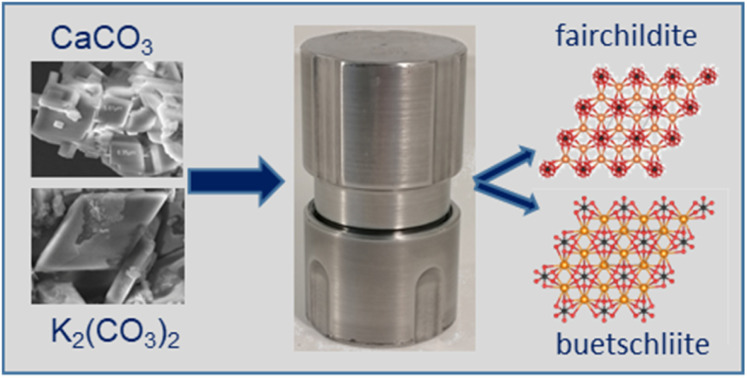

## Introduction

Since the initial work of Le Chatelier^1^ and Niggli,^2^ the K_2_CO_3_–CaCO_3_ system has been the subject of numerous phase analytical studies.^3–6^ Cooper *et al.* identified two binary compounds with the following K_2_CO_3_ : CaCO_3_ ratios in air at atmospheric pressure: 1 : 2 (or K_2_Ca_2_(CO_3_)_3_) and 1 : 1 (or K_2_Ca(CO_3_)_2_).^5^ The 1 : 2 phase exhibits incongruent melting behavior, with a melting point of *T*_L_ = 810 °C, and decomposes below 512 °C into K_2_Ca(CO_3_)_2_ and CaCO_3_ (calcite). Meanwhile, K_2_Ca(CO_3_)_2_ melts congruently at 809 °C. The polymorphic nature of K_2_Ca(CO_3_)_2_ was first reported by Mrose *et al.*, identifying a transformation point (*T*_c_) at 704 °C.^7^ The high- and low-temperature modifications correspond to synthetic analogues of the minerals fairchildite and buetschliite, respectively.^8^ Cooper *et al.* and Pabst confirmed the existence of a temperature-induced phase transition but determined considerably lower values for *T*_c_.^5,9^ Their reported transformation temperatures are consistent with each other, ranging between 505–585 °C (ref. 9) and 547 °C.^5^ The enthalpies and entropies of formation for both polymorphs of K_2_Ca(CO_3_)_2_ and for K_2_Ca_2_(CO_3_)_3_ were measured by drop calorimetry.^10^

Studies on the synthesis of fairchildite from carbonates in air through solid-state reactions revealed that the synthesis temperature must be above 585 °C and the sample needs to be quenched.^9^ Although no further details were given on the kinetics of the reverse reaction, a spontaneous back-transformation to buetschliite was observed, albeit slowly. The phase transformation is first order and reconstructive. Further details on the structural aspects of this transformation can be found later in this section. Both modifications are highly soluble in water and susceptible to alteration in a humid atmosphere. However, it can be inferred that both compounds are relatively stable under natural conditions since they occur as minerals on the Earth's surface. Pabst proposed that an initial reaction with water might create a protective CaCO_3_ layer, which would passivate the surface.^9^

For a long time, double carbonates from the binary system K_2_CO_3_–CaCO_3_ were considered rare in nature. The aforementioned minerals buetschliite and fairchildite were first observed in the 1930s and 1940s in ‘clinkers’ found in the trunks of partially burnt conifers such as fir or hemlock, for example. These solid masses were formed by melting and subsequent crystallization of the corresponding wood ashes, likely triggered by lightning strikes.^8^ Occurrences of these so-called fused wood-ash stones can be found in the western states of the USA and Canada.^11^ Fairchildite's chemical formula K_2_Ca(CO_3_)_2_ was correctly assigned the from the beginning,^8^ while the correct composition of buetschliite, initially assumed to be a hydrous carbonate, was finally determined by Mrose *et al.*, clarifying that the two compounds are polymorphs.^7^

The significance of K_2_Ca(CO_3_)_2_ in mineralogy, particularly petrology, has increased with the discovery of these phases in magmatic rocks. For instance, Sharygin *et al.* reported the occurrence of fairchildite in multiphase inclusions in magnetites from the phoscorites of the Loolekop deposit within the Phalaborwa alkali carbonatite complex (South Africa).^12^ Buetschliite was discovered alongside eitelite (Na_2_Mg(CO_3_)_2_) and dolomite as inclusions in type Ia diamonds from the Sytykanskaya kimberlite pipe in Yakutia (Russia).^13^ The authors interpreted this paragenesis as a quench product of a former carbonate melt under mantle conditions, providing insight into the composition of the environment in which the diamonds crystallized. Buetschliite is nowadays considered a crucial compound for comprehending the carbon cycle in the Earth's mantle.

Consequently, the stability of K_2_Ca(CO_3_)_2_ was experimentally investigated in recent years at high pressures using multi-anvil systems as well as diamond anvil cells. Experimental series conducted at 3, 6, and 19 GPa indicate that buetschliite remains stable up to approximately 6 GPa before undergoing a structural phase transition with symmetry reduction, evidenced by a splitting of characteristic bands in the Raman spectra.^14–17^ Additionally, an increase in temperature from ambient conditions to 300 °C resulted in a shift of the pressure-induced transformation to approximately 8 GPa.^16^

Fairchildite and buetschliite are found not only in geosciences but also in materials related to applied mineralogy or industrial inorganic chemistry. For instance, fairchildite has been identified in biochar fertilizers produced through plasma pyrolysis from nutrient-rich biomass residues or effluent sludge waste.^18^ Additionally, it has been observed that fairchildite can also be found in biomass combustion ashes.^19,20^ This is not that unexpected given the natural formation conditions described earlier. It is also noteworthy that K_2_Ca(CO_3_)_2_ is present as a phase in a sustainable heterogeneous catalyst material used for biodiesel synthesis, derived from plant leaves.^21^

**Fig. 1:**
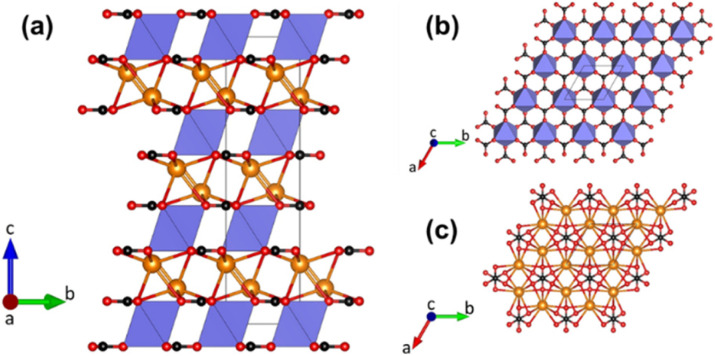
(a) Projection of the crystal structure of buetschliite along [100] based on the structural data of Effenberger and Langhof.^23^ Calcium atoms are coordinated by six nearest oxygen neighbours from adjacent carbonate groups in form of octahedra (shown in light blue). Potassium atoms (yellow) are surrounded by nine oxygen ligands. Oxygen and carbon atoms are presented as red and black spheres, respectively. (b) Top view of a single layer (*z* ≈ 0) of CaO_6_-octahedra and planar carbonates groups. (c) Top view of a single layer (*z* ≈ ⅙) containing K-cations and planar carbonate groups.

Buetschliite crystallises in the trigonal space group *R*3̄2/*m* with *a* = 5.38 Å, *c* = 18.12 Å, and *Z* = 3.^9^ Fairchildite, on the other hand, has the following basic crystallographic data: space group *P*6_3_/*mmc*, *a* = 5.294 Å, *c* = 13.355 Å, and *Z* = 2.^22^ Buetschliite does not exhibit any orientational disorder of the carbonate groups (Fig. 1) and there is no mixed occupation of the K and Ca ions on the two cation positions, which have coordination numbers of 6 (in the case of calcium) or (6 + 3) (in the case of potassium).

**Fig. 2:**
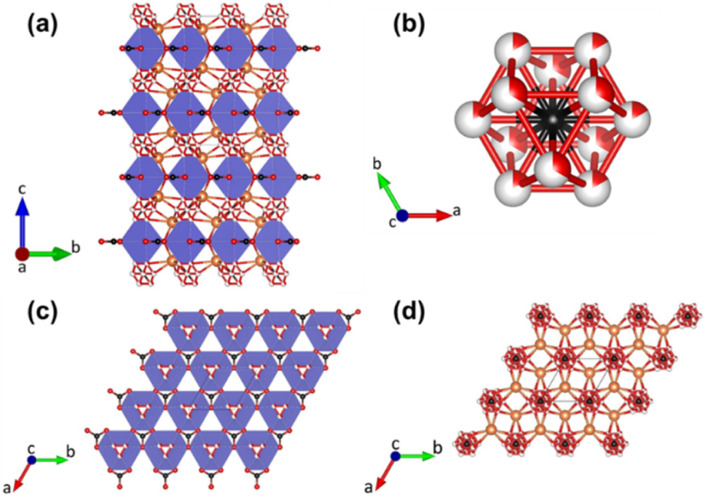
(a) Projection of the crystal structure of fairchildite along [100]. Calcium dominated sites are coordinated by 12 oxygen atoms (light blue polyhedron). Potassium dominated positions are shown in yellow. Oxygen and carbon atoms are presented as red and black spheres, respectively. A part of the carbonate groups exhibits a pronounced disorder. Bicolored spheres indicate the corresponding partially occupied oxygen positions. The sizes of the bicolored segments refer to the percentages from the site-occupancy refinements as determined by Pertlik.^22^ (b) Distribution of the six possible disordered positions of carbonate groups around the carbon atom in (0, 0, 0). The partially occupied oxygen atoms define the 12 corners of a cube-octahedron. (c) Top view of a single layer (*z* ≈ ¼) containing the CaO_12_-polyhedra and the non-disordered carbonate groups. (d) Top view of a single layer (*z* ≈ 0) containing the K-enriched sites and the strongly disordered carbonate moieties.

The high-temperature phase fairchildite exhibits a significantly higher degree of disorder, with 50% of the carbonate groups being disordered (Fig. 2). In addition, the two cation species, K and Ca, are statistically distributed over two sites and coordinated by twelve and fifteen oxygen ions, respectively, if disordered oxygens from the carbonate groups are counted separately. The higher degree of structural disorder in fairchildite is also reflected in higher entropy values in thermochemical measurements, as reported by Navrotsky *et al.* in 1997.^10^

When studying the ternary system K_2_O–CaO–SiO_2_ using silica and the two carbonates K_2_CO_3_ and CaCO_3_ as precursors, we observed the formation of mixed cation carbonates upon homogenizing the reactants in a planetary ball mill at 600 rpm for 45 min. Ethanol was used as the milling fluid. After milling, the slurries were dried at 60 °C for 12 hours to fully evaporate the alcohol. The dry precursors obtained were then analysed by powder X-ray diffraction prior to commencing the high-temperature solid-state reactions. Interestingly, qualitative phase analysis consistently revealed the presence of the synthetic analogue to the mineral buetschliite along with the three starting reagents. This observation suggests that a mechanochemical reaction occurred between K_2_CO_3_ and CaCO_3_, resulting in the formation of K_2_Ca(CO_3_)_2_. Such a mechanochemically triggered conversion between carbonates has never been reported yet. Therefore, we performed further investigation. The aim was to reveal the formation process of the mixed carbonates under milling conditions and to understand the role of ethanol and water in that process as water is present as crystal water and ethanol is a commonly used milling agent in liquid assisted grinding (LAG).

This work reports the results of detailed *ex situ* laboratory studies and *in situ* synchrotron X-ray powder diffraction experiments. The latter enable the investigation of mechanochemical reactions occurring inside a jar during operation in a Retsch MM400 shaker mill.

## Experimental

### Chemicals and milling equipment

Calcium carbonate (CaCO_3_) and potassium carbonate sesquihydrate (K_2_CO_3_·1.5H_2_O) with a purity of ≥99.0% were purchased from Sigma-Aldrich. To investigate the influence of crystal water and/or moisture the mechanochemical transformation, the chemicals were used once dried and once undried. For the undried samples, CaCO_3_ and K_2_CO_3_·1.5H_2_O were weighed in stoichiometric amounts (1 : 1) without taking the crystal water of potassium carbonate into account. In a second experiment, both salts were dried at 300 °C for 10 hours, which is more than 200 °C higher than the dehydration to anhydrous potassium carbonate.^24^ After drying, the starting materials were transferred as quickly as possible to glass vials and placed in a glove box where they were weighed into the milling jars.

All syntheses were carried out with a MM400 shaker-mill from Retsch. Stainless steel milling jars with a volume of 25 mL and two 15 mm stainless steel balls (13.6091 (±0.0056) g each) from the same company were used for all experiments. For the reaction 0.5870 (±0.0002) g potassium carbonate and 0.4130 (±0.0002) g of calcium carbonate were used (1 g in total). The samples were milled with different frequencies, *i.e.*, 20, 25, and 30 Hz. In addition, the milling time was varied between 30 and 120 min.

### *Ex situ* X-ray powder diffraction experiments

After milling, the samples were filled into borosilicate glass capillaries with a wall thickness of 0.01 mm and an inner diameter of 0.5 mm. The capillaries were sealed with wax to avoid any contact with moisture. The data were collected with a STADI P transmission diffractometer from STOE & Cie GmbH. The instrument used monochromatic MoKα_1_ radiation (*λ* = 0.7093 Å) and a Mythen 1K detector. Data were recorded in the range between 3 and 50° 2*θ* with a step size of 0.015° 2*θ*. The measurement time per step was 20 s. Data were also collected under ambient conditions on a X-Pert Pro diffractometer (PANanytical) in Bragg–Brentano geometry, CuKα_1,2_ radiation and a position sensitive X'Celerator detector. The crystal structure data for Rietveld analysis were taken from the ICSD database, version 5.2.0, data release 2024.1: CaCO_3_ (20 179),^25^ K_2_CO_3_·1.5H_2_O (22 257),^26^ K_2_CO_3_ (66 943),^27^ KHCO_3_ (2074),^28^ fairchildite (100 845),^22^ and buetschliite (29 442).^23^ Numbers refer to the entry codes of the ICSD. Quantitative phase analysis was performed with TOPAS V6 (Bruker AXS GmbH, Karlsruhe, Germany).^29^ Quantification was based on crystal structure data of known compounds, one of which having a distorted structure. Atomic positions were not refined, only scaling factors and lattice parameters were refined. The Rietveld plots, residuals and quantification are given in the ESI.† Figures showing structural features were prepared using the program VESTA3.^30^

### *In situ* X-ray powder diffraction experiments

*In situ* X-ray diffraction data were recorded at DESY (Hamburg), PETRAIII, beamline P02.1. The data were collected with a Varex XRD 4343CT detector using a wavelength of 0.207352 Å and a counting rate of 60 s per frame. Data integration was performed with the software package Dawn 2.6.0 (Diamond Light Source Ltd, Oxfordshire, United Kingdom).^31^ For the experiments, a commercial shaker mill (Retsch MM400) with modified clamping system and jars was mounted on the diffractometer. Information about the general experimental setting and all modifications can be found in the paper published by Rathmann *et al.*^32^

## Results

### *Ex situ* X-ray powder diffraction studies

#### Variation of milling frequencies using undried educts

**Fig. 3:**
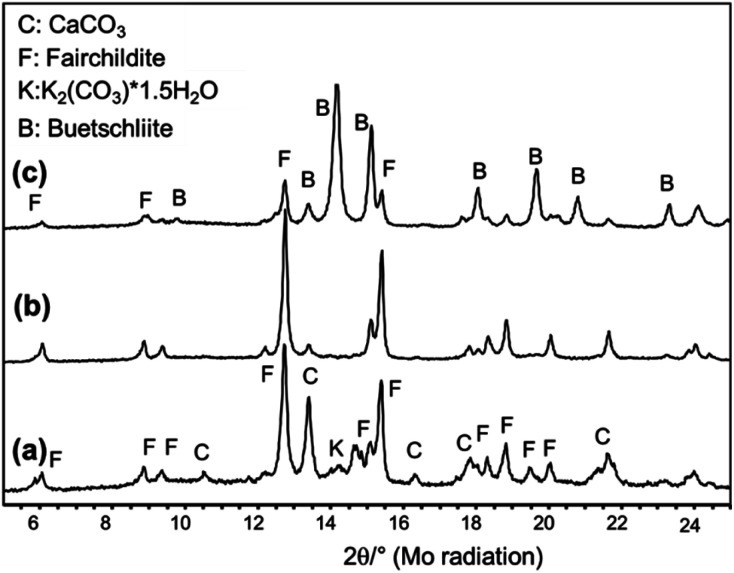
*Ex situ* X-ray powder patterns of the undried educts collected after milling for 60 min at (a) 20 Hz, (b) 25 Hz, and (c) 30 Hz.

In the first series of experiments, the educts CaCO_3_ and K_2_CO_3_·1.5H_2_O were not dried before milling allowing the influence of moisture and structural water of potassium carbonate on the process to be studied. All educt mixtures were milled at shaking frequencies of 20, 25, and 30 Hz. After the milling experiments, the samples were filled into glass capillaries, sealed, and XRD data were collected immediately after the experiments. Fig. 3 shows the XRD patterns collected after 60 min of milling at different frequencies. At 20 Hz, fairchildite has formed but the conversion of the educts is not complete (Fig. 3a).

The reaction of the educt carbonates to fairchildite is completed after 60 min when the milling frequency is increased to 25 Hz (Fig. 3b). Only about 6 wt% CaCO_3_ is left from the educts. A further increase to 30 Hz promotes the formation of buetschliite as the main phase (76 wt%) alongside fairchildite (23 wt%), and less than 1 wt% CaCO_3_ (Fig. 3c).

**Fig. 4:**
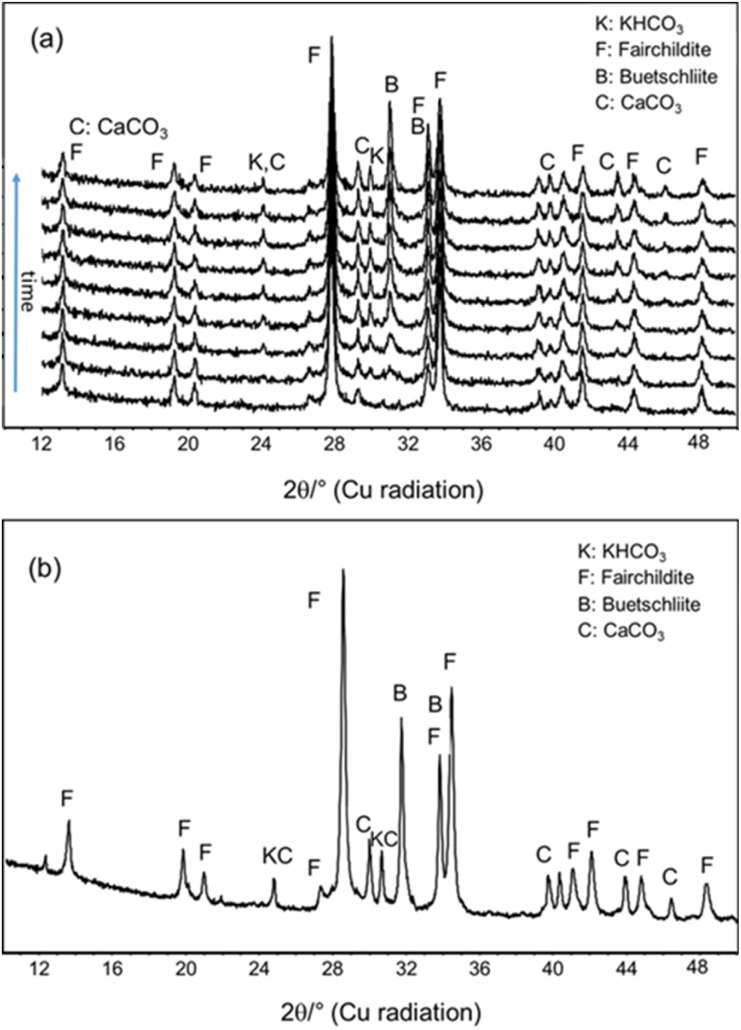
(a) *Ex situ* X-ray powder patterns of the sample obtained after ball milling at 25 Hz for 60 min of undried educts: bottom data were measured after milling and then every 24 h (192 h total time, upper pattern) under ambient conditions. Intensities are plotted on absolute scale with an offset between the datasets. (b) Final powder pattern collected after 192 exposure of the sample to ambient conditions.

Parallel to these studies, the sample milled for 60 min at 25 Hz shown in Fig. 3b was prepared for X-ray powder diffraction measurements under ambient conditions to study the influence of storage time (Fig. 4a). Continuously collected data show the gradual transformation of fairchildite into buetscheliite over time. Quantitative Rietveld analysis of the powder pattern collected after 192 h reveals that already about 21 wt% buetschliite have formed beside 66 wt% fairchildite, 4 wt% CaCO_3_ and 9 wt% KHCO_3_ (Fig. 4b).

The error of the quantitative Rietveld refinements is considered to be about 1–2 wt% as the CaCO_3_ is strongly textured. The educt mixture contains a surplus amount of 5.7 wt% of CaCO_3_. The phase amount of residual CaCO_3_ at 25 Hz of 6 wt% thus indicates that, within the error range of the Rietveld refinements, likely no K_2_Ca_2_(CO_3_)_3_ is formed.

#### Variation of milling frequencies using dried educts

Next, the educts were dried at 300 °C for 10 h and afterwards filled in a glovebox into the milling jars.

**Fig. 5:**
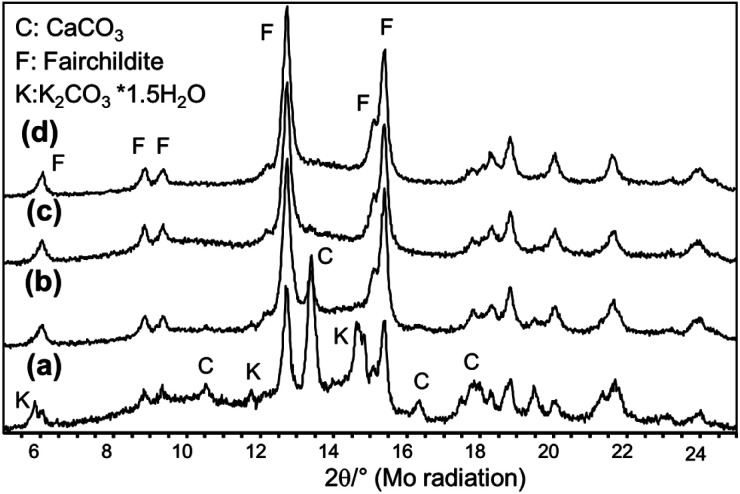
*Ex situ* X-ray powder patterns obtained after milling of dried educts for (a) 60 min at 25 Hz, (b) 60 min at 30 Hz, (c) 90 min at 30 Hz, and (d) 120 min at 30 Hz. Intensities are plotted on an absolute scale with an offset between the datasets.

After milling for 60 min with 25 Hz, only a small amount of fairchildite has formed while the main fraction of the starting materials has not reacted (Fig. 5a). The increase of the frequency to 30 Hz leads to a higher conversion of the starting mixture into the double carbonate (Fig. 5b) and prolongation of the milling times to 90 min further increases the amount of fairchildite (Fig. 5c). After 120 min the reaction is completed (Fig. 5d). In contrast to the non-dried starting materials that transformed into buetschliite after 60 min at 30 Hz, the absence of crystal water or moisture seems to suppress the transformation of fairchildite to buetschliite.

### *In situ* X-ray powder diffraction studies

#### Variation of milling frequencies using undried educts

**Fig. 6:**
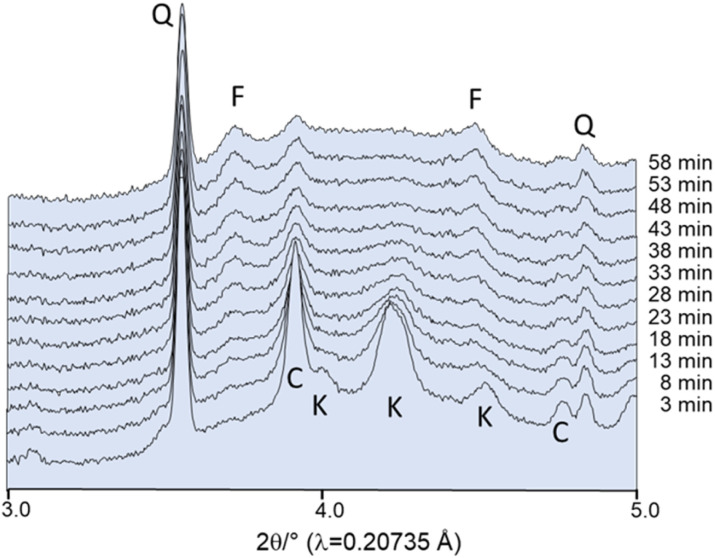
*In situ* X-ray powder pattern collected during milling at 25 Hz: F: fairchildite, C: CaCO_3_, K: K_2_CO_3_, Q: quartz. Intensities are plotted on a square root scale with an offset between the datasets.

**Fig. 7:**
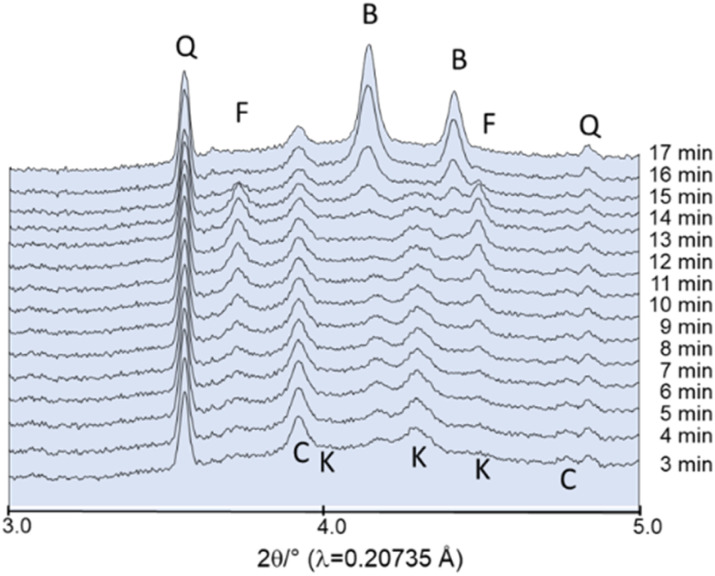
*In situ* powder pattern collected during milling at 27 Hz: F: fairchildite, C: CaCO_3_, B: buetschliite, K: K_2_CO_3_, Q: quartz. Intensities are plotted on a square root scale with an offset between the datasets.

In this experiment, the starting materials were not subjected to drying prior to milling. As the initial experiments demonstrated that the powder adheres to the walls of the milling jar (Fig. S1†), a small quantity of crystalline quartz was introduced to the reaction. Quartz is chemically inert and functions as a milling medium, removing the powder from the walls. This increases the accessibility of the powder to the X-ray beam, thereby enhancing the data quality. The first powder pattern shown in Fig. 6 was collected after 3 min of milling. Potassium carbonate sesquihydrate has lost the crystal water and the reflections can be assigned to water-free K_2_CO_3_ (*P*2_1_/*c*). The formation of fairchildite starts already after about 20 min milling at 25 Hz. In accordance with the *ex situ* experiments, even after 60 min milling unreacted CaCO_3_ can be observed and fairchildite has not transformed to buetschliite (Fig. 6). *In situ* studies allow to facilitate the modification of milling conditions and monitor directly how the changes affect the reaction. A modest rise in frequency from 25 to 27 Hz provides sufficient energy input for the educts to react to fairchildite within the first few min and to buetschliite after approximately 15 min (Fig. 7).

#### Influence of absolute EtOH using dried educts

**Fig. 8:**
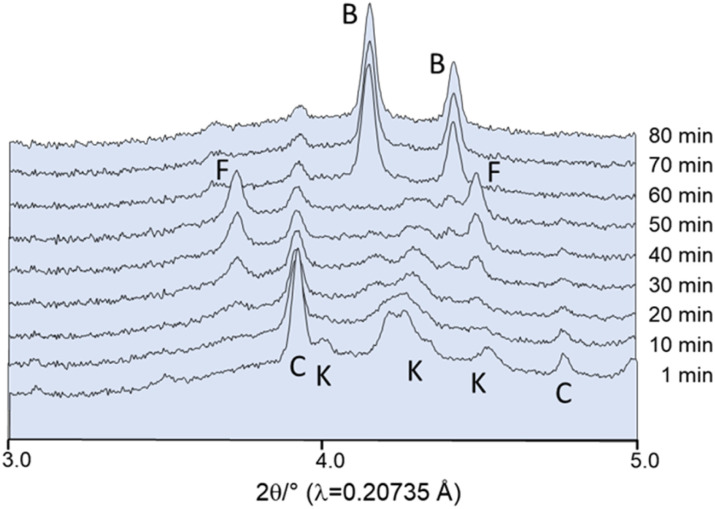
*In situ* powder patterns collected during milling at 25 Hz. Before milling, 10 drops of absolute ethanol have been added to the jar. F: fairchildite, C: CaCO_3_, B: buetschliite, K: K_2_CO_3_. Intensities are plotted on a square root scale with an offset between the datasets.

For the following *in situ* time-dependent synchrotron diffraction experiments, the starting materials were dried prior to the reaction. An amount of 10 drops of ethanol (about 200 μL, corresponding to *η* = 200 μL/1000 mg = 0.2 μL mg^−1^)^33^ was added right before milling at 25 Hz to study the influence of solvents on the reaction. Already after 30 min, fairchildite is formed which is completely converted to buetschliite after 60 min (Fig. 8).

**Fig. 9:**
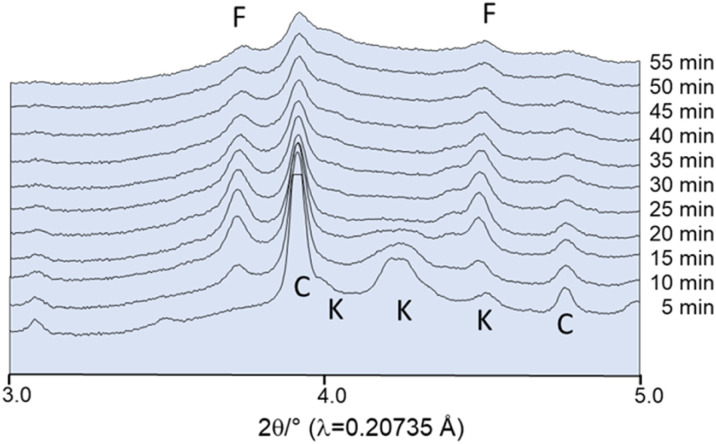
*In situ* powder patterns collected after milling of dried educts at 25 Hz. F: fairchildite, C: CaCO_3_, K: K_2_CO_3_. Intensities are plotted on a square root scale with an offset between the datasets.

In contrast, the milling of the dried compounds without the addition of EtOH does indeed result in the formation of fairchildite. However, even after 55 min of milling, the reaction is not yet complete, and the educts can still be observed (Fig. 9).

The reduction in the overall pattern intensities with extended milling times is due to the adherence of the powder to the jar caps, which results in the powder being outside the X-ray beam's focal point. No further transformation to the thermodynamically stable buetschliite was observed within the first 60 min of milling. However, upon storing the fairchildite sample in the laboratory, the transformation to the thermodynamically stable buetschliite proceeds unsystematically. The storage conditions, the presence of humidity, and different particle sizes seem to play an important role.

## Discussion

The studies have shown that CaCO_3_ and K_2_CO_3_ can react under mechanochemical conditions to form the double salts fairchildite and buetschliite. Notably, in all experiments, the structurally disordered high-temperature polymorph, fairchildite, forms first. This observation follows Ostwald's rule of stages.^34^ Based on this rule, crystallisation proceeds to equilibrium from an initial high-energy state through changes in the free energy. Therefore, the less stable fairchildite should be formed first before transforming to the stable buetschliite.

Previously, it was reported that the synthesis of fairchildite from carbonates in air through solid-state reactions requires temperatures above 585 °C for several days followed by subsequent quenching. However, our studies prove that the high-temperature polymorph can also be synthesised *via* mechanochemical reaction without any external heating and within less than one hour. Depending on the milling conditions, fairchildite remains stable for at least 120 min or transforms into the structurally ordered, low-temperature polymorph buetschliite.

Buetschliite has been discussed to be the stable polymorph under high pressures up to 6 GPa, while fairchildite is stable at lower pressures.^14^ Applying pressures between 0.1 and 3 GPa to fairchildite leads to the transformation to buetschliite. Past studies have repeatedly reported that very high temperatures are generated during milling as a result of the impact of the balls on the samples. The latter would explain the formation of the high-temperature polymorph fairchildite. However, this explanation does not suffice for the transformation of fairchildite into buetschliite, which usually occurs slowly under ambient conditions. The theory of hot spot generation upon ball milling assumes the impact of a milling ball on a single crystallite. Such an impact indeed might generate very high temperature, as the impact of a milling ball will expose its force onto a very small crystal surface. However, under average milling conditions, the ball will hit into a polycrystalline ensemble of agglomerated particles. Thus, the impact will be into a powder layer rather than onto a single crystallite. The temperature increase thus will be moderate whereas the pressure exerted onto the powder in the impact volume will be significant. Moreover, studies have shown that the temperatures inside a shaker mill do not exceed 100 °C.^35,36^ Therefore, our findings suggest that the influence of pressure, rather than high temperatures, is the reason for the formation of buetschliite. The effect of pressure has been also assumed to drive the formation of calcite to aragonite upon ball milling.^37–39^

Frequency has been demonstrated to be a crucial factor that decides whether the reaction leads to the formation of the disordered high-temperature phase fairchildite or the ordered thermodynamically stable buetschliite. The higher the shaking frequency, the higher the kinetic energy of the milling balls and, as the consequence, the higher the pressure generated by the impact will be. The energy needs to be high enough to initiate the specific reactions/transformations. In our experiments using a shaker mill with 1 g of sample in 25 mL jars, 20 Hz resulted in low conversions of the educts, while higher frequencies such as 25 Hz led to a full conversion to fairchildite and 30 Hz even promoted the transformation to buetschliite.

Another factor that appears to be important for such types of reactions is the presence of moisture. While the formation of fairchildite is not significantly affected by the presence of crystal water, the transformation of fairchildite to buetschliite does not proceed in the absence of water even at high grinding frequencies of 30 Hz and extended grinding times of up to 120 min. The amount of 1.5H_2_O molecules per formula unit of K_2_CO_3_ accounts for about 9.6 wt% and accelerates the formation of buetschliite, as shown in the *in situ* XRD experiments. A similar effect is observed when small amounts of ethanol are added to dried products. As water and ethanol are both polar molecules that can dissolve at least K_2_CO_3_ to some extent, mobilization of K^+^ and CO_3_^2−^ in a thin liquid layer of water on the crystal surfaces may facilitate the transformation. A similar effect is discussed for rock salt, which creeps under pressure in the presence of minimal amounts of water likely *via* a dissolution-precipitation process. Longo and Voight discussed that a thin water film on the surface could enhance the mechanochemical reaction of carbonates.^40^ Recently, Jiang *et al.* studied the influence of water on the solubility of carbonates and the mechanochemical synthesis of dolomite.^41^

While liquid assisted grinding (LAG) has become of interest for mechanochemical reactions of soft materials in the last twenty years,^42^ the role of water (mostly surface water or crystal water) for inorganic reactions has been known for a long time. The influence of water molecules and/or OH groups in general on mechanochemical processes of inorganic materials has been intensively studied for various inorganic reactions.^43–45^ The role of water molecules is discussed to be manifold as water seems to promote the diffusion through a liquid phase on the surface of the milled particles and the formation of highly active surface groups.^43^ With respect to the role of water in the formation of the double carbonates discussed here, future *in situ* investigations, *e.g.*, based on *in situ* IR or Raman spectroscopy, may shed more light on this specific reaction. However, this is beyond the scope of this paper.

## Conclusions

In this paper we report the synthesis of both K_2_Ca(CO_3_)_2_ polymorphs fairchildite and buetschliite from the carbonates CaCO_3_ and K_2_CO_3_ by mechanochemical reaction in a shaker mill. Milling of the educts leads in all cases first to the formation of the high-temperature polymorph fairchildite. Depending on the milling parameters, further transformation to the low-temperature polymorph buetschliite can take place. The variation of the milling frequency and the presence of moisture/solvents govern the reaction and enable the directed synthesis of the polymorphs without any additional temperature input or pressure treatment.

## Data availability

The authors confirm that the data supporting the findings of this study are available within the article and the ESI† (Rietveld refinements).

## Conflicts of interest

There are no conflicts to declare.

## Supplementary Material

MR-002-D4MR00093E-s001
