# Supplementary material for: Mechanochemical synthesis and transformation of the polymorphic double carbonates fairchildite and buetschliite, (K2Ca(CO3)2): an in situ X-ray powder diffraction study
Source: RSC Mechanochem. 2024 Nov 28;2(1):152–8. doi: 10.1039/d4mr00093e (PMC11603408; doi:10.1039/d4mr00093e)

This journal is © The Royal Society of Chemistry 2024

Supporting Information

Mechanochemical synthesis and transformation of the polymorphic double carbonates fairchildite and buetschliite, ($K_2Ca(CO_3)_2$): an in situ X-ray powder diffraction study.

Volker Kahlenberg[a],*, Doris E. Braun[b], Wolfgang Schmidt[c], Hang Liu[a], Sebastian Leiting[c], Claudia Weidenthaler[c]*

The experimental details are given in the main text: All XRD measurements were performed in transmission geometry (capillary optics) using Mo-radiation, a primary monochromator and a Mythen detector.

Quantitative phase analysis was performed using the program package TOPASV6. Only the scaling factors, lattice parameters, and the peak shape parameters have been refined. The background was refined using a Chebychev function of 5th order. Crystal structure data were used from the ICSD database as listed in the main text. Data were refined in the range between 1 and 30°2θ.

(a) Educts were not dried before milling. 20 Hz, 60 min, R_{wp} 8.7%, GOF: 1.8

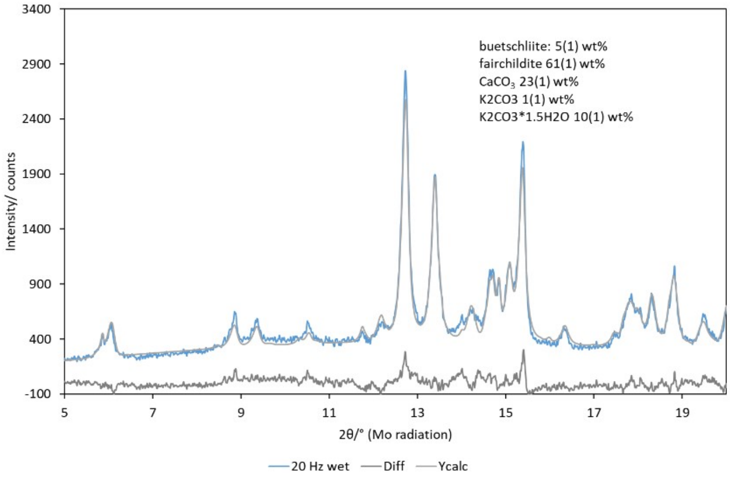

(b) Educts were not dried before milling. 25 Hz, 60 min, R_{wp}: 11.4%, GOF:2.5

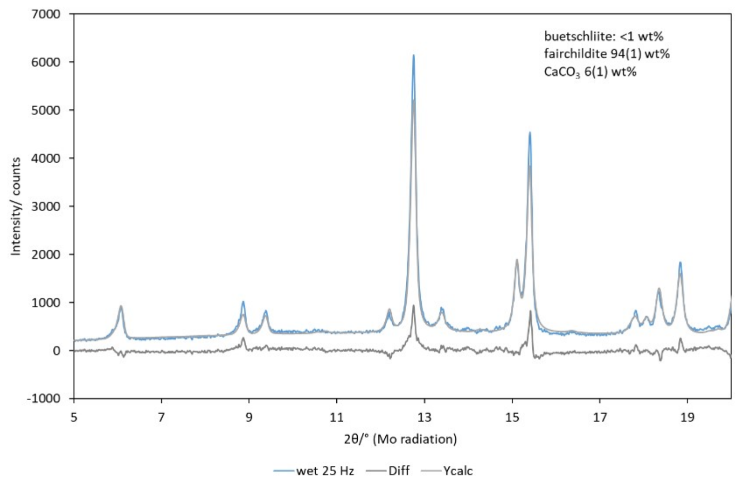

(c) Educts were not dried before milling. 30 Hz, 60 min, R_{wp}: 10.0%, GOF: 2

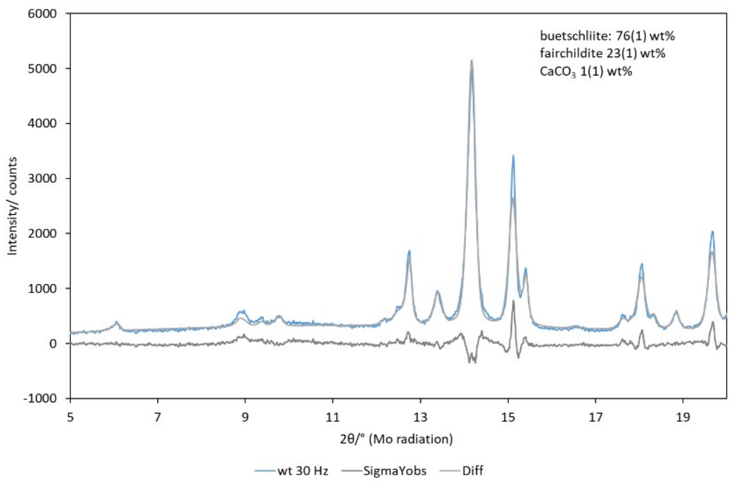

The following Rietveld plot shows the data after 192 h continuous measurement (Figure 4b, main text). Data were collected in Bragg-Brentano geometry with Cu radiation. R_{wp}: 10.9%, GOF: 1.1

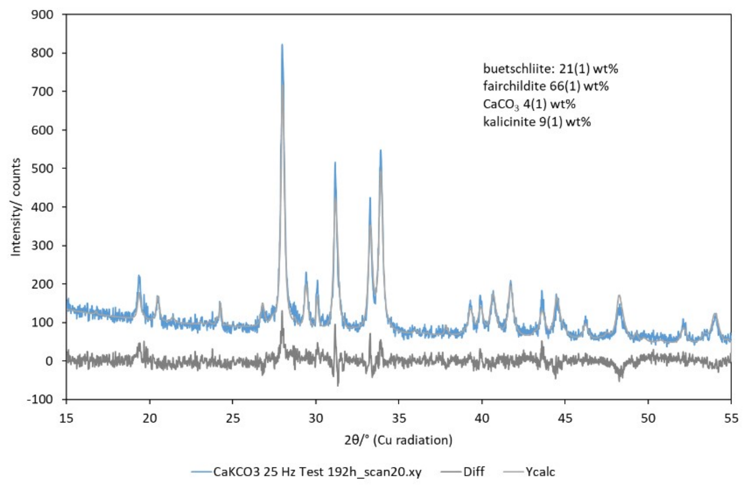

Supplement: MR-002-D4MR00093E-s001 [file MR-002-D4MR00093E-s001.pdf]
